# Sagittal intervertebral rotational motion: a deep learning-based measurement on flexion–neutral–extension cervical lateral radiographs

**DOI:** 10.1186/s12891-022-05927-0

**Published:** 2022-11-08

**Authors:** Yuting Yan, Xinsheng Zhang, Yu Meng, Qiang Shen, Linyang He, Guohua Cheng, Xiangyang Gong

**Affiliations:** 1 The Second School of Clinical Medicine, Zhejiang Chinese Medical University, Hangzhou, 310053 Zhejiang China; 2 Rehabilitation Medicine Center, Department of Radiology, Zhejiang Provincial People’s Hospital, Affiliated People’s Hospital, Hangzhou Medical College, Hangzhou, 310014 Zhejiang China; 3 Hangzhou Jianpei Technology Company Ltd, Hangzhou, 311200 Zhejiang China; 4 Institute of Artificial Intelligence and Remote Imaging, Hangzhou Medical College, Hangzhou, 310014 Zhejiang China

**Keywords:** Deep learning, Automated measurement, Cervical spine, Motion analysis, Radiography

## Abstract

**Background:**

The analysis of sagittal intervertebral rotational motion (SIRM) can provide important information for the evaluation of cervical diseases. Deep learning has been widely used in spinal parameter measurements, however, there are few investigations on spinal motion analysis. The purpose of this study is to develop a deep learning-based model for fully automated measurement of SIRM based on flexion–neutral–extension cervical lateral radiographs and to evaluate its applicability for the flexion–extension (F/E), flexion–neutral (F/N), and neutral–extension (N/E) motion analysis.

**Methods:**

A total of 2796 flexion, neutral, and extension cervical lateral radiographs from 932 patients were analyzed. Radiographs from 100 patients were randomly selected as the test set, and those from the remaining 832 patients were used for training and validation. Landmarks were annotated for measuring SIRM at five segments from C2/3 to C6/7 on F/E, F/N, and N/E motion. High-Resolution Net (HRNet) was used as the main structure to train the landmark detection network. Landmark performance was assessed according to the percentage of correct key points (PCK) and mean of the percentage of correct key points (MPCK). Measurement performance was evaluated by intra-class correlation coefficient (ICC), Pearson correlation coefficient, mean absolute error (MAE), root mean square error (RMSE), and Bland-Altman plots.

**Results:**

At a 2-mm distance threshold, the PCK for the model ranged from 94 to 100%. Compared with the reference standards, the model showed high accuracy for SIRM measurements for all segments on F/E and F/N motion. On N/E motion, the model provided reliable measurements from C3/4 to C6/7, but not C2/3. Compared with the radiologists’ measurements, the model showed similar performance to the radiologists.

**Conclusions:**

The developed model can automatically measure SIRM on flexion–neutral–extension cervical lateral radiographs and showed comparable performance with radiologists. It may provide rapid, accurate, and comprehensive information for cervical motion analysis.

**Supplementary Information:**

The online version contains supplementary material available at 10.1186/s12891-022-05927-0.

## Background

Cervical kinematics evaluation plays an indispensable role in cervical-related diseases, including neck pain, whiplash-associated disorders, and cervical instability [1–3]. As the main part of cervical intervertebral motion parameters, sagittal intervertebral rotational motion (SIRM) provides precise biomechanical information and reveals early abnormal motion patterns [4, 5].

Originally, intervertebral motion parameters were measured manually with fine pencils on X-ray films [6]. Such handwork resulted in significant observer differences, which accounted for 38% or more of the value being measured [7]. Computer-assisted methods and various software have been developed for landmark location, parameter measurement, and data analysis to minimize subjective influence and accelerate processing speed [8–10]. However, manual annotation of the landmarks on vertebral bodies was unavoidable in these methods, which brought tedious and time-consuming work for operators.

Deep learning has been increasingly applied to the measurement of musculoskeletal radiographs [11–13]. In the field of spinal disorders, a variety of models have demonstrated good to excellent performance in automatically measuring Cobb angle, spinopelvic parameters, and sagittal alignment, with the mean absolute error (MAE) ranging from 1° to 5° [14–17]. Aside from static spinal parameter measurements, there were some studies focusing on the deep learning-based measurement for intervertebral motion. Jacobsen et al. [18] acquired cervical intervertebral angles on fluoroscopic images with a landmark detection algorism for cervical joint movement evaluation. Nguyen et al. [19] developed a deep learning system to measure lumbar intervertebral angles based on flexion and extension radiographs in order to determine the instability of the lumbar spondylolisthesis. The above studies have some limitations in using static parameters measured from a separate view to explore spinal motion function. Consequently, it is necessary to carry out a measurement derived from the combination of multiple views, which may provide more accurate and comprehensive information for spinal motion analysis.

The objective of the present study was to develop a fully automated deep learning model for the measurement of SIRM based on flexion–neutral–extension cervical lateral radiographs and to evaluate its applicability for the flexion–extension (F/E), flexion–neutral (F/N), and neutral–extension (N/E) motion analysis.

## Methods

### Dataset preparation

**Fig. 1:**
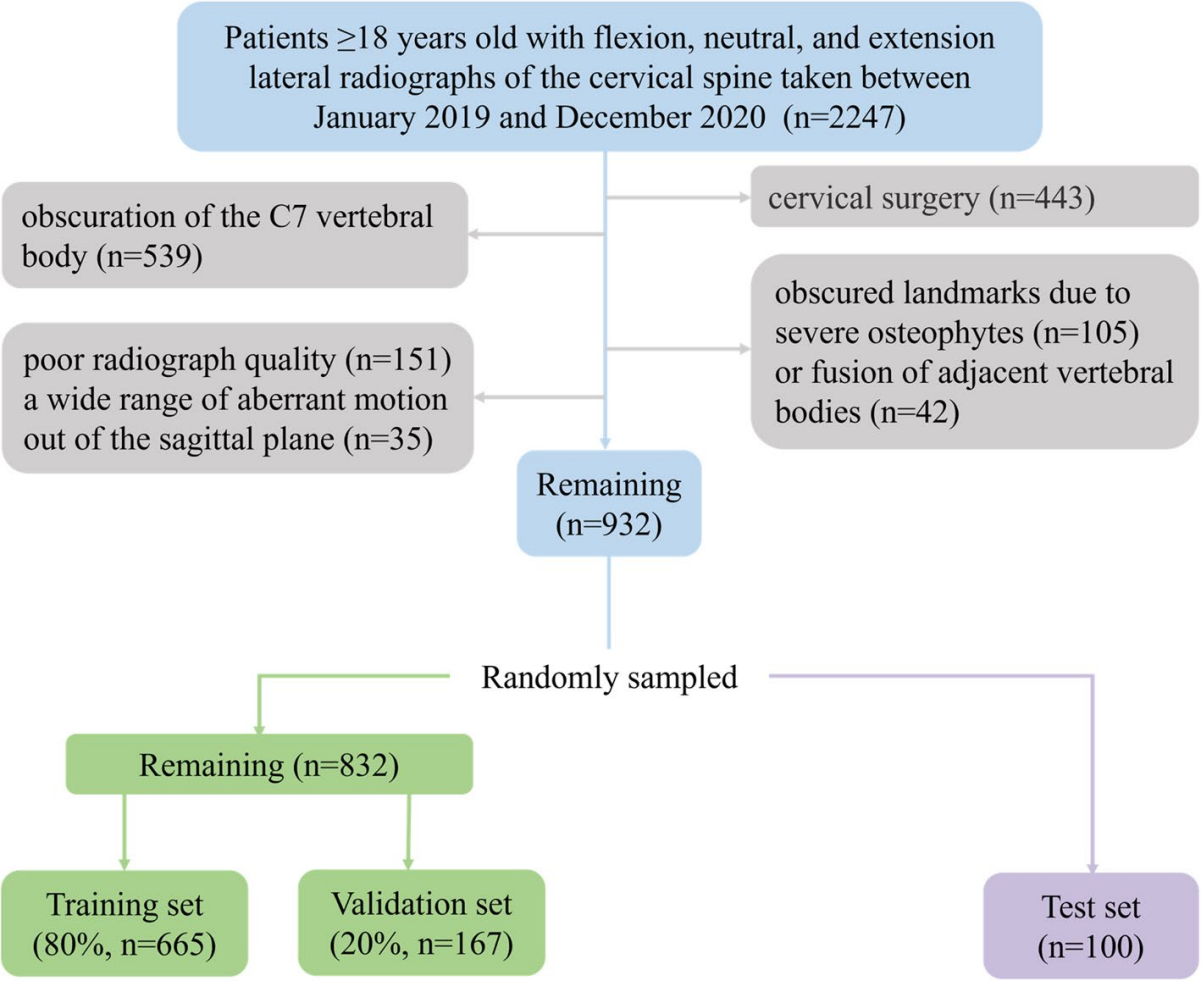
Flowchart of the inclusion criteria and dataset distribution for the training, validation, and test sets

This study was approved by the Institutional Research Ethics Committee of Hospital (2022QT041). A total of 2247 cases for which flexion, neutral, and extension cervical lateral radiographs were taken in inpatient and outpatient settings between January 2019 and December 2020 were reviewed. The data of these patients were sequentially collected from the Picture Archiving and Communication System (PACS) of hospital. Adult patients (≥18 years) were included to ensure skeletal maturity in all cases in this study. The need for informed consent was waived due to the use of retrospective data. The exclusion criteria were as follows: (1) a history of cervical surgery; (2) partial or complete obscuration of the C7 vertebral body; (3) obscured landmarks due to severe osteophytes or fusion of adjacent vertebral bodies; and (4) poor radiograph quality and a wide range of aberrant motion out of the sagittal plane. From this review, a total of 2796 cervical lateral radiographs from 932 patients were collected. Radiographs from 100 cases were randomly assigned to the test set, and those from the remaining 832 cases were randomly divided in a 4:1 ratio to form the training and validation sets, respectively (Fig. 1).

### Landmark annotations

The training and validation sets were manually annotated by one musculoskeletal radiologist (R1, 5 years of experience), and 100 cases of them were reannotated by R1 after a 6-week interval for the assessment of intra-observer reliability. The test set was independently annotated by R1, together with R2 (a radiologist with 3 years of experience) and R3 (a radiologist with 5 years of experience). All radiologists discussed and agreed on the annotation method before starting the work. A dedicated website was developed for manual annotation (http://warehouse.healthviewcn.com/).

### Definitions of landmarks and parameter

**Fig. 2:**
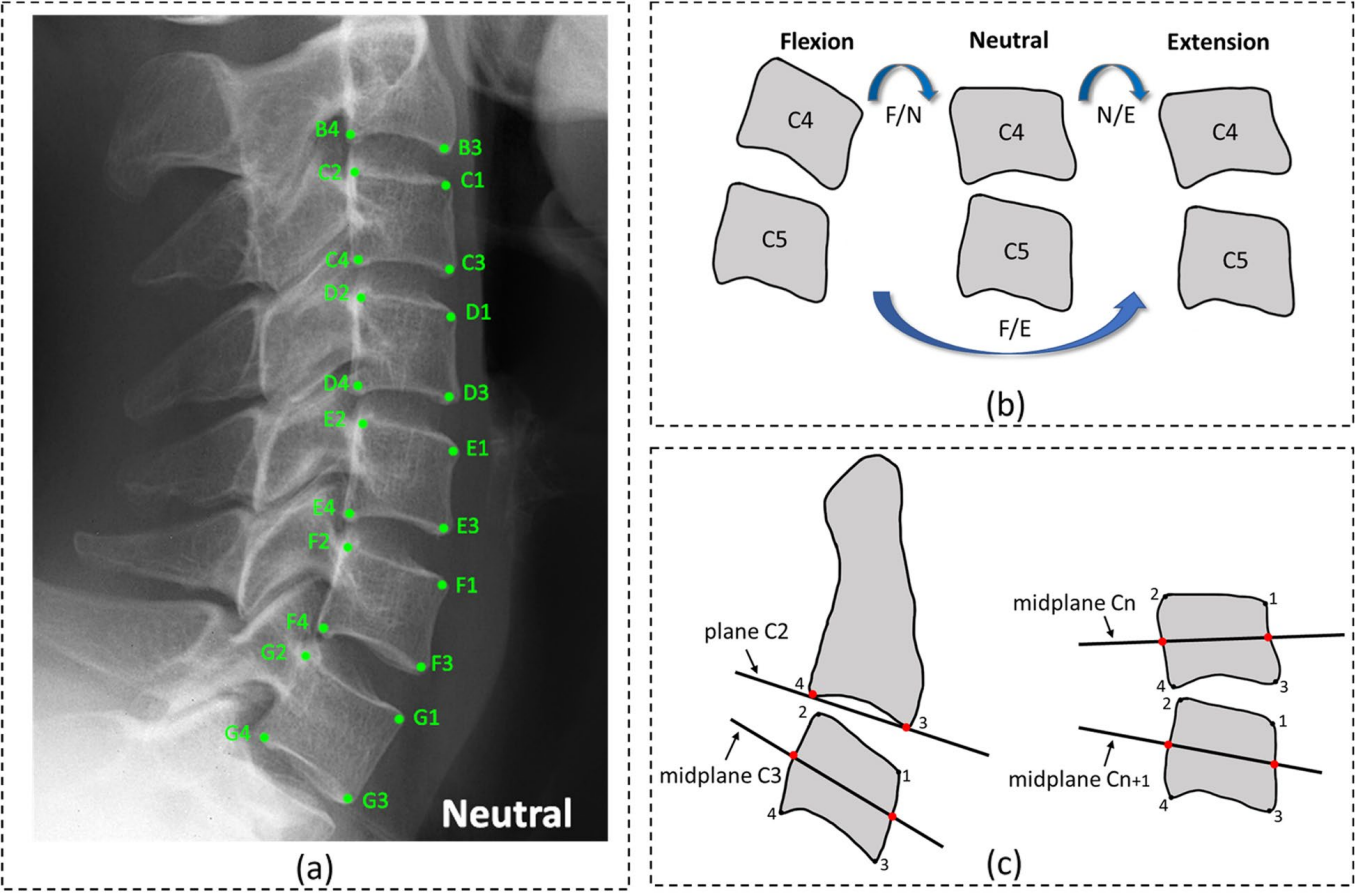
Landmark annotations and the method for sagittal intervertebral rotational motion (SIRM) measurement. **a** Annotations of landmarks (taking the neutral view as an example). Each landmark has a specific name. **b** Sagittal intervertebral motion ranges included flexion–extension (F/E), flexion–neutral (F/N), and neutral–extension (N/E) motion based on flexion, neutral, and extension views (taking C4/5 as an example). **c** Measurement method for SIRM. For C2/3 (left), the SIRM was the difference in the angles between the plane of C2 and the midplane of C3 on F/E, F/N, and N/E motion. For C3/4-C6/7 (right), the SIRM was the difference in the angles between the midplane of Cn and Cn + 1 (3 ≤ n ≤ 6) on F/E, F/N, and N/E motion

A total of 22 landmarks were annotated on each radiograph on the flexion, neutral, and extension views. For the typical vertebrae from C3 to C7, the anterior–superior, posterior–superior, anterior–inferior, and posterior–inferior vertebral body corners were denoted by C-G1 to C-G4, respectively. For C2 vertebrae with a unique biological shape, only the anterior–inferior and posterior–inferior corners were denoted by B3 and B4, respectively. To reduce measurement error, all annotations of landmarks were made as close to the corticomedullary margin of the vertebral body as possible [7]. The method for measuring SIRM was based on the geometric midplanes method, for which excellent agreement and smaller errors have been demonstrated [20, 21]. The vertebral midplane was defined by a line through the two midpoints between the anterior and posterior corners. The specific name of each landmark and the method for measurement are illustrated in Fig. 2.

### Measurement model development

The deep learning model for SIRM measurement included two parts: a landmark detection network used to identify landmarks on flexion, neutral, and extension cervical lateral radiographs and mathematical formulae to calculate SIRM values.

The main structure of the landmark detection network was High-Resolution Net (HRNet), a novel deep convolutional neural network with excellent performance in localizing anatomical landmarks on medical images [22]. HRNet maintains high-resolution representations through parallel branches from beginning to end and repeatedly fuses features from different scales to achieve solid semantics and accurate location. Due to the proximity of the landmarks on cervical radiographs, HRNet’s ability to preserve image details is crucial for model training. The landmark detection network consisted of four stages, beginning with a high-resolution branch as the first stage and then summing the high-to-low resolution branches in parallel to form the subsequent stages. At the end of each stage, information was repeatedly exchanged between parallel branches [23]. The final output was a 22-channel heatmap regressed from the high-resolution representation of the last stage. The coordinates with the maximum values in the heatmap were selected as the positions of predicted landmarks, which were mapped to the corresponding positions on the original image by applying affine transformation. To compare the ground truth and prediction heatmaps, the loss function was defined as the mean square error.

To build the landmark detection network, we trained our model on flexion, neutral, and extension cervical lateral radiographs. All images were preprocessed by resizing to a resolution of 512 × 512 pixels and augmented by random rotation, horizon flip, and random scale. The pixel spacing of each image was 0.143 mm. We used the stochastic gradient descent (SGD) optimizer with a base learning rate of 1e^− 6^, a momentum of 0.9, and a weight decay of 0.0005. The model was trained on PyTorch (Version 1.3) for 120 iterations with a batch size of 12 on NVIDIA TITAN Xp GPUs. The model with the least loss on the validation set was verified using the test set.

**Fig. 3:**
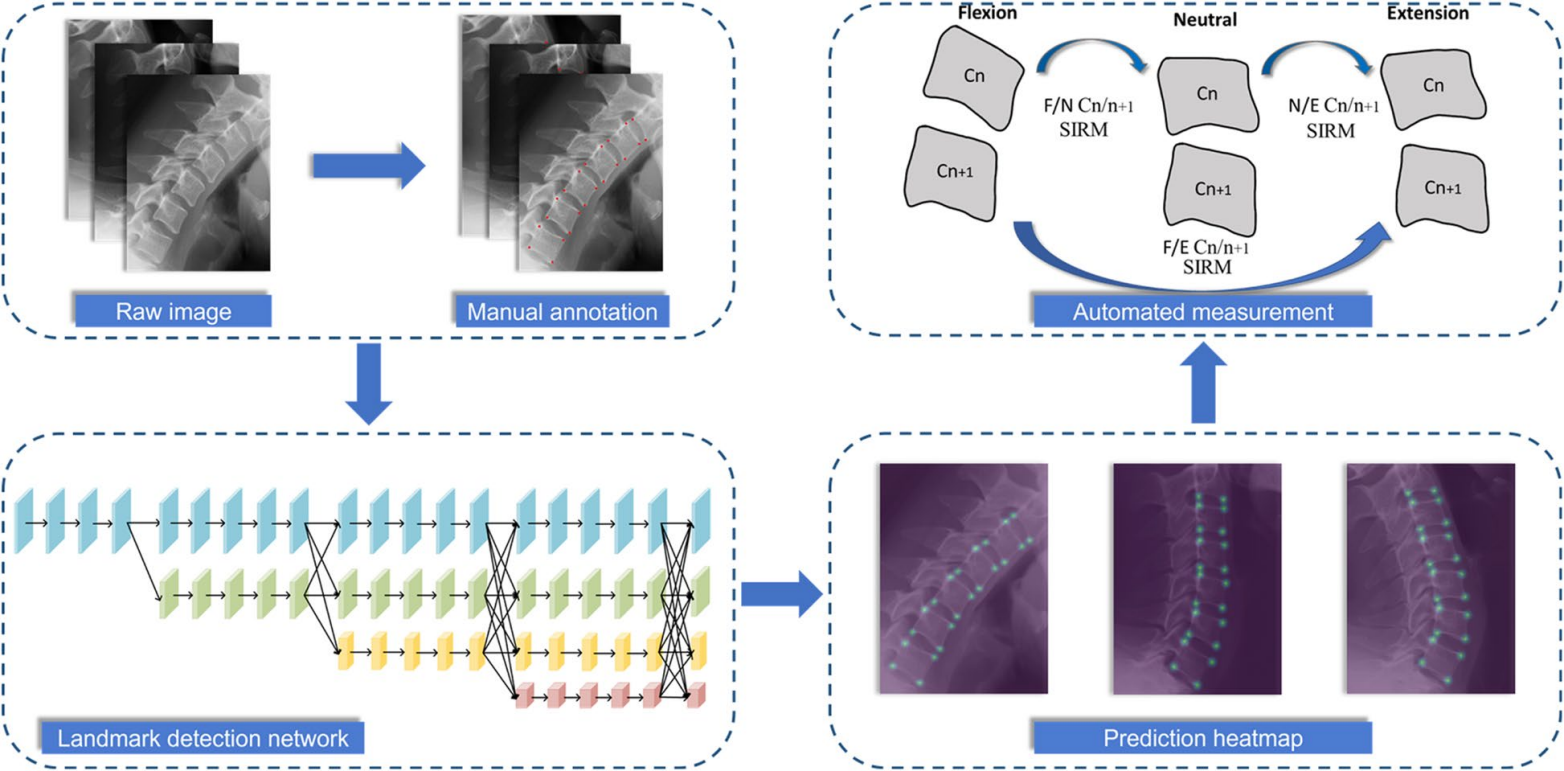
Overview of model implementation. SIRM: sagittal intervertebral rotational motion

The coordinates of the predicted landmarks and the mathematical formulae for measurement were used for automatic calculations of SIRM by Python (version 3.7). An overview of model implementation is presented in Fig. 3.

### Evaluation and statistical analysis

All analyses were performed using MedCalc software (version 20.023) and Microsoft Excel 2020 with statistical significance defined by values of *P* < 0.05.

### Reliability of landmark annotation

For assessment of the intra- and inter- observer reliability of landmark annotation, the landmark-to-landmark distance was represented as percentages within 1–3-mm thresholds.

### Landmark performance

The metrics of percentage of correct key points (PCK) and mean of the percentage of correct key points (MPCK) were used for evaluating the performance of landmark prediction. PCK was defined as the percentage of predicted landmarks that fell within the r-radius neighborhood of the reference standard landmark [24]. MPCK was defined as the mean PCK values of each vertebra from C2 to C7 (MC2–MC7) [22]. The reference standards were the averages of landmark coordinates annotated by the three radiologists [25].

### Measurement performance

For further evaluation of measurement performance, our model was compared with the reference standards on the test set by calculating the intra-class correlation coefficient (ICC), the Pearson correlation coefficient (r), MAE, and the root mean square error (RMSE). The ICC was calculated for the evaluation of consistency, and an ICC ≥0.7 was considered adequate for reliability. An |r| ≥0.7 indicated a high correlation. MAE was defined as $\frac{1}{m}{\sum}_{i=1}^m\left|{observed}_i-{predicted}_i\right|$, and RMSE was defined as $\sqrt{\frac{1}{m}{\sum}_{i=1}^m{\left({observed}_i-{predicted}_i\right)}^2}$, where i was the number of images. Additionally, the mean difference and 95% limit of agreement (LoA) were determined on Bland–Altman plots. The reference standards were the averages of measurements from the three radiologists [25]. To compare the performance of the model with that of each radiologist, the paired differences between the value from each individual radiologist and the average from the other two radiologists were compared with the difference between the same average value and the model value using paired t-test for comparing MAE [12].

## Results

### General data distributions

A total of 932 cases with flexion, neutral, and extension cervical lateral radiographs were evaluated. There were 665, 167, and 100 patients in the training, validation, and test sets, respectively. There was no significant difference between the three datasets in gender composition and age (Table 1).

**Table 1:** Characteristics of patients in the training, validation, and test sets

Characteristic	Training set (*n* = 665)	Validation set (*n* = 167)	Test set (*n* = 100)	P
Male	283 (42.6)	59 (35.3)	42 (42)	0.234
Female	382 (57.4)	108 (64.7)	58 (58)	
Age(y)^a^	49 (47,50)	49 (46,52)	49 (45,54)	0.646
Male	47 (45,49)	49 (43,54)	47 (38.5,52.5)	0.836
Female	50 (48,51)	49 (46,53)	52.5 (45,58)	0.326

Data are expressed as numbers of patients, with percentages in parentheses

*P* < 0.05 indicates significant difference between the training, validation, and test sets

^a^Data are expressed as medians, with 95% confidence intervals (CI) in parentheses

### Reliability of landmark annotation

The percentages of intra-observer landmark distances within the 2-mm threshold were 98–99% on the three views. The percentages of inter-observer landmark distances within the 2-mm threshold were 97–98% (R1 vs R2), 98% (R1 vs R3), and 97–98% (R2 vs R3) on the three views (Table 2).

**Table 2:** Inter-observer reliability of landmark annotation (%) on flexion, neutral, and extension views

Threshold		1 mm			2 mm			3 mm	
	F	N	E	F	N	E	F	N	E
R1 vs R2	84	85	84	97	98	97	99	99	99
R1 vs R3	85	86	85	98	98	98	99	99	99
R2 vs R3	79	81	81	98	98	97	99	99	99

*F* Flexion, *N* Neutral, *E* Extension

### Landmark performance

**Fig. 4:**
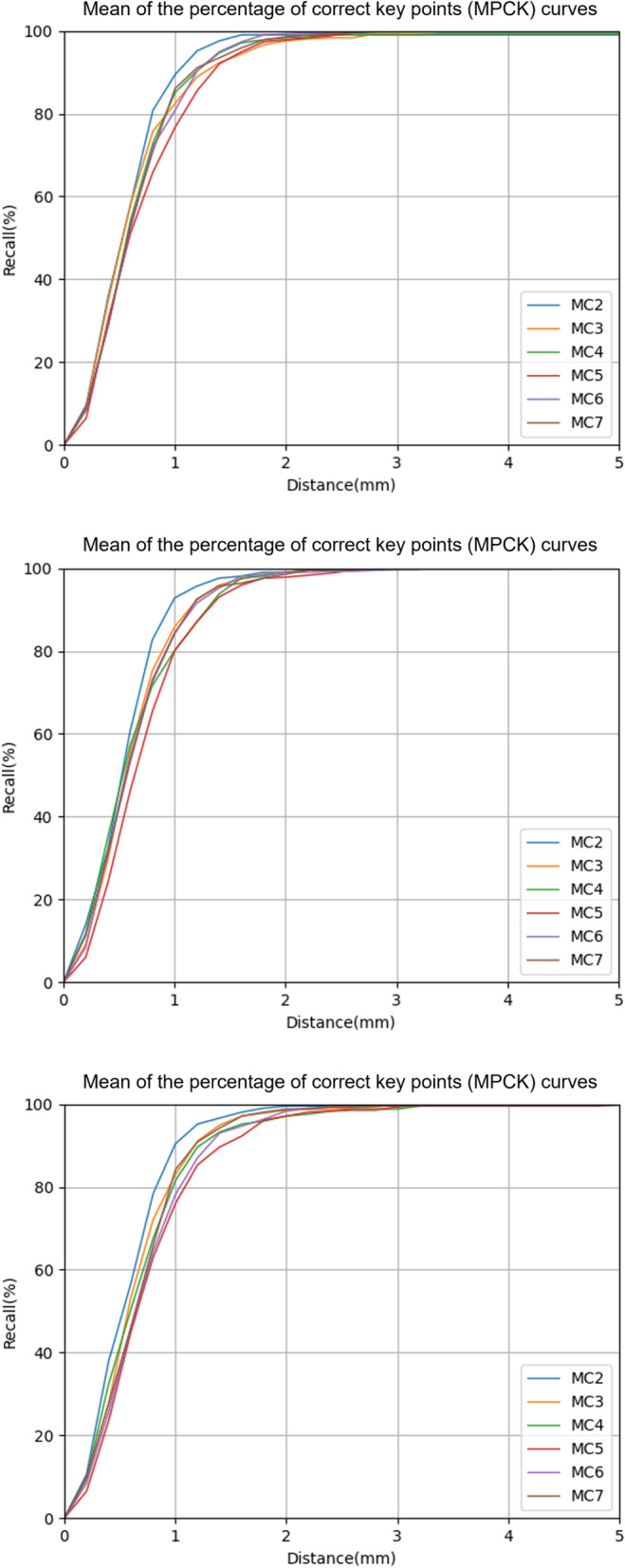
The ability of landmark prediction. The prediction ability of our model is shown for each vertebra from C2 to C7. The first to third rows show the performance on flexion, neutral, and extension views, respectively

**Fig. 5:**
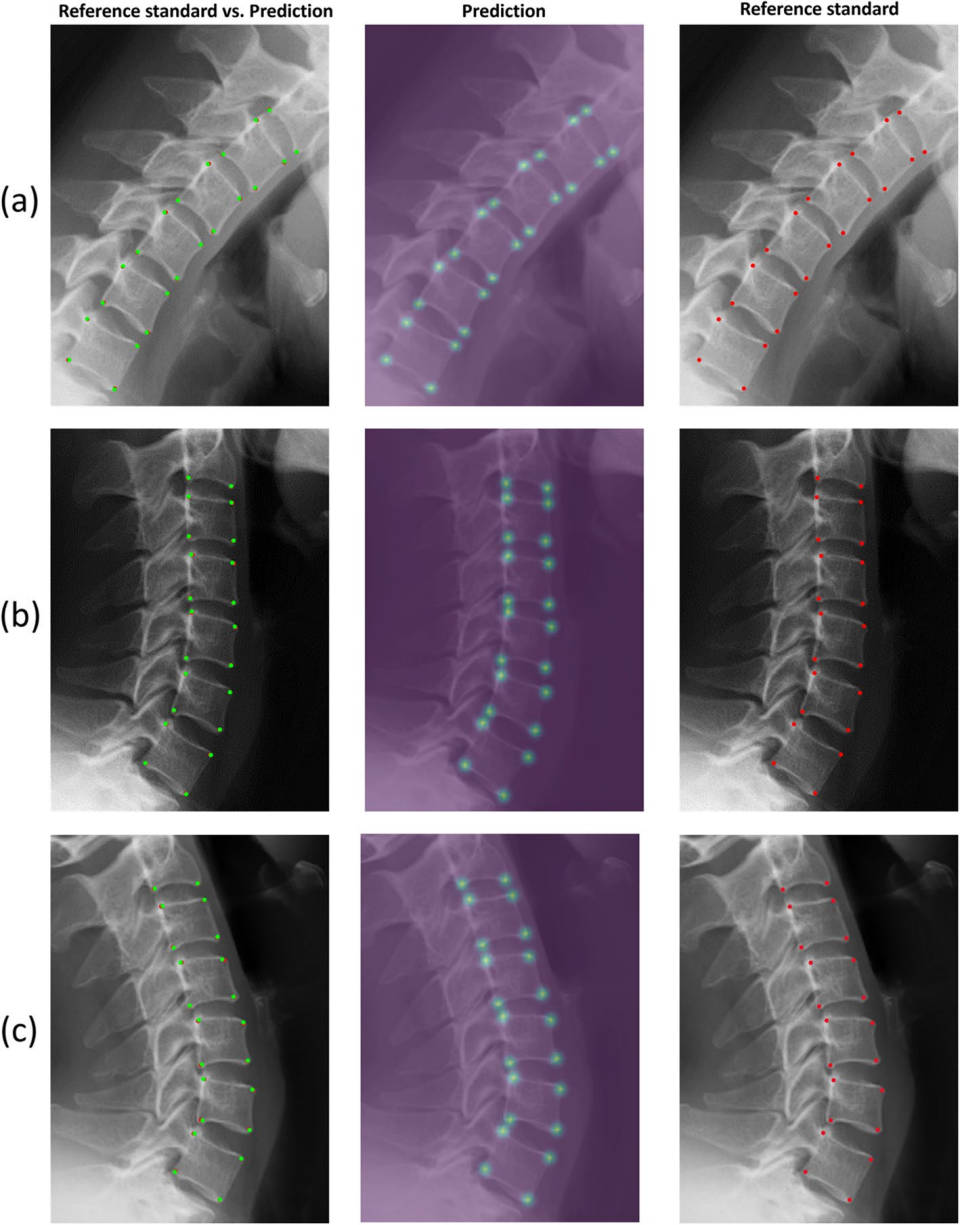
Representative images illustrating landmark detection by our model. **a**, **b**, and **c** are examples of flexion, neutral, and extension views, respectively

The PCKs at the 2-mm distance threshold on the flexion, neutral, and extension views were 95–100%, 94–100%, and 94–100%, respectively (Additional file 1). The MPCKs for each vertebra from C2 to C7 at the 2-mm distance threshold on the flexion, neutral, and extension views were 98–99%, 98–99%, and 97–100%, respectively (Fig. 4). The average annotating time for one cervical lateral radiograph was 0.066 s, which was much faster than the annotating time of 2.1 min for a radiologist. Representative examples of landmark detection by the model are shown in Fig. 5.

### Measurement performance

Measurement values from three radiologists and model estimates for SIRM were shown in Table 3. No significant differences were observed in the SIRM produced by the model estimates and the reference standards, except for the values for C2/3 (F/E motion), C3/4 (F/N motion), and C3/4 (N/E motion) (*P* < 0.05).

**Table 3:** Measurement values from three radiologists and model estimates for sagittal intervertebral rotational motion (SIRM) (°)

	R1	R2	R3	Mean	Model	t	P
F/E motion							
C2/3	5.07 ± 3.36	6.19 ± 3.32	5.29 ± 3.33	5.52 ± 2.98	5.01 ± 3.29	−3.327	0.001
C3/4	10.95 ± 4.81	10.46 ± 4.72	10.82 ± 4.61	10.74 ± 4.49	10.78 ± 4.77	0.218	0.827
C4/5	12.40 ± 5.36	12.56 ± 5.63	12.66 ± 5.35	12.54 ± 5.23	12.68 ± 5.66	0.683	0.496
C5/6	11.46 ± 6.17	11.39 ± 5.93	11.44 ± 6.10	11.43 ± 5.90	11.21 ± 6.23	−1.028	0.307
C6/7	9.84 ± 5.18	9.73 ± 5.17	9.85 ± 5.05	9.81 ± 4.95	9.59 ± 5.21	−1.311	0.193
F/N motion							
C2/3	3.71 ± 2.71	4.02 ± 2.64	3.87 ± 2.60	3.87 ± 2.19	3.94 ± 2.64	−0.392	0.696
C3/4	6.13 ± 3.95	6.36 ± 3.39	6.22 ± 3.89	6.24 ± 3.49	6.79 ± 3.73	−3.118	0.002
C4/5	6.96 ± 4.08	6.98 ± 4.09	7.20 ± 3.71	7.05 ± 3.69	7.22 ± 4.29	0.748	0.456
C5/6	7.55 ± 4.28	7.49 ± 4.25	7.47 ± 4.38	7.50 ± 4.08	7.32 ± 4.51	−0.881	0.381
C6/7	7.86 ± 4.39	7.72 ± 4.15	7.80 ± 4.22	7.79 ± 4.01	7.65 ± 3.93	−0.952	0.344
N/E motion							
C2/3	2.84 ± 2.29	3.00 ± 2.37	2.77 ± 2.07	2.87 ± 1.74	2.69 ± 1.88	−1.095	0.276
C3/4	5.06 ± 3.80	4.38 ± 3.63	4.76 ± 3.69	4.73 ± 3.47	4.27 ± 3.39	−2.789	0.006
C4/5	5.91 ± 4.11	5.81 ± 4.05	5.57 ± 3.95	5.76 ± 3.84	5.64 ± 4.04	−0.585	0.560
C5/6	4.57 ± 3.51	4.25 ± 2.94	4.47 ± 3.25	4.43 ± 2.93	4.65 ± 3.38	1.127	0.262
C6/7	2.71 ± 2.09	2.62 ± 2.21	2.76 ± 2.36	2.70 ± 1.90	2.72 ± 2.41	0.123	0.902

Data are expressed as the means ± SDs

*F/E* Flexion–extension, *F/N* Flexion–neutral, *N/E* Neutral–extension

*P* < 0.05 (paired t-test) indicates significant difference between the model and reference standard

**Fig. 6:**
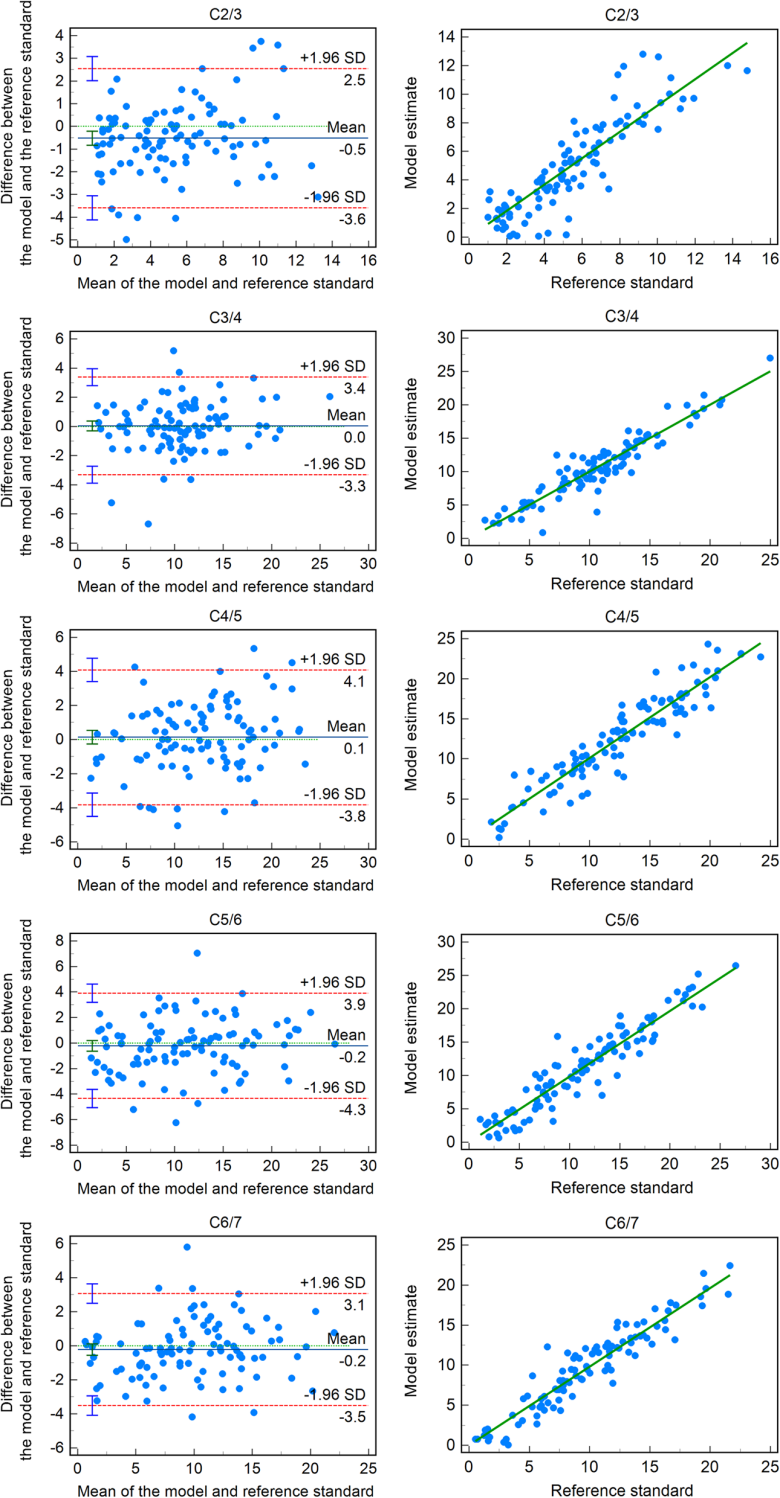
Bland–Altman plots (left) and correlation scatter diagrams (right) showing the differences and correlations between the model and reference standards on flexion–extension (F/E) motion

With regard to consistency and accuracy, the model yielded accurate measurements of all segments on F/E motion (ICC = 0.86–0.95, *r* = 0.88–0.95, RMSE = 1.64–2.11, MAE = 1.22–1.59) and F/N motion (ICC = 0.73–0.93, *r* = 0.74–0.93, RMSE = 1.50–2.27, MAE = 1.19–1.69). On N/E motion, the model provided reliable measurements from C3/4 to C6/7 (ICC = 0.73–0.88, *r* = 0.75–0.88, RMSE = 1.59–2.05, MAE = 1.28–1.49), but not C2/3 (ICC = 0.60, *r* = 0.61, RMSE = 1.61, MAE = 1.21; Table 4). The Bland–Altman plots with 95% LoAs and scatter diagrams of correlation analysis are shown in Fig. 6 (F/E motion) and Additional files 2 and 3 (F/N and N/E motion).

**Table 4:** Comparison of model estimates and reference standards for sagittal intervertebral rotational motion (°)

	ICC (95% CI)	r (95% CI)	Mean Difference	SD	RMSE (95% CI)	MAE (95% CI)
F/E motion						
C2/3	0.86 (0.79, 0.91)	0.88 (0.83,0.92)	−0.52	1.57	1.64 (1.44,1.90)	1.23 (1.01,1.45)
C3/4	0.93 (0.90,0.95)	0.93 (0.90, 0.96)	0.04	1.70	1.70 (1.49,1.97)	1.22 (0.99,1.46)
C4/5	0.93 (0.90,0.95)	0.93 (0.90, 0.96)	0.14	2.02	2.01 (1.77,2.33)	1.57 (1.32,1.82)
C5/6	0.94 (0.91, 0.96)	0.94 (0.91, 0.96)	−0.22	2.10	2.11 (1.85,2.45)	1.59 (1.32,1.87)
C6/7	0.95 (0.92, 0.96)	0.95 (0.92, 0.96)	−0.22	1.68	1.69 (1.48,1.96)	1.29 (1.08,1.51)
F/N motion						
C2/3	0.73 (0.62, 0.81)	0.74 (0.64, 0.82)	0.07	1.79	1.78 (1.56,2.07)	1.40 (1.18,1.62)
C3/4	0.87 (0.81, 0.92)	0.88 (0.83, 0.92)	0.55	1.75	1.83 (1.61,2.12)	1.37 (1.13,1.61)
C4/5	0.84 (0.77, 0.89)	0.85 (0.78, 0.90)	0.17	2.27	2.27 (1.99,2.63)	1.69 (1.39,1.99)
C5/6	0.87 (0.84, 0.92)	0.89 (0.84, 0.93)	−0.18	2.05	2.05 (1.80,2.38)	1.66 (1.42,1.90)
C6/7	0.93 (0.90, 0.95)	0.93 (0.90, 0.95)	−0.14	1.50	1.50 (1.32,1.74)	1.19 (1.00,1.37)
N/E motion						
C2/3	0.60 (0.46, 0.71)	0.61 (0.46, 0.72)	−0.18	1.61	1.61 (1.41,1.87)	1.21 (1.00,1.43)
C3/4	0.88 (0.82, 0.92)	0.88 (0.83, 0.92)	−0.46	1.66	1.71 (1.50,1.98)	1.34 (1.13,1.55)
C4/5	0.87 (0.81, 0.91)	0.87 (0.81, 0.91)	−0.12	2.05	2.05 (1.80,2.38)	1.49 (1.21,1.77)
C5/6	0.80 (0.72, 0.86)	0.81 (0.73, 0.87)	0.22	1.98	1.99 (1.75,2.31)	1.43 (1.16,1.71)
C6/7	0.73 (0.62, 0.81)	0.75 (0.65, 0.82)	0.02	1.60	1.59 (1.40,1.85)	1.28 (1.10,1.47)

*ICC* Intra-class correlation coefficient, *r* Pearson correlation coefficient, *SD* Standard deviation, *RMSE* Root mean square error, *MAE* Mean absolute error, *CI* Confidence interval, *F/E* Flexion–extension, *F/N* Flexion–neutral, *N/E* Neutral–extension

On F/E motion, the MAEs of the model were significantly lower than those of R1 and R2 at C2/3 (*P* < 0.05) and those of R2 at C3/4 and C6/7 (*P* < 0.05). On F/N motion, the MAEs of the model were significantly lower than that of R3 at C2/3 (*P* < 0.05) and those of R1, R2, and R3 at C6/7 (*P* < 0.05). On N/E motion, the MAE of the model was significantly lower than that of R1 at C2/3 (*P* < 0.05; Table 5).

**Table 5:** Comparisons of each radiologist and the model for sagittal intervertebral rotational motion

MAE	Mean of R2 and R3			Mean of R1 and R3			Mean of R1 and R2		
	F/E	F/N	N/E	F/E	F/N	N/E	F/E	F/N	N/E
C2/3									
Radiologist*	1.92	1.67	1.70	1.95	1.71	1.63	1.78	1.86	1.59
Model	1.33	1.55	1.26	1.30	1.56	1.33	1.54	1.49	1.37
P	0.00	0.48	0.01	0.00	0.36	0.06	0.09	0.04	0.18
C3/4									
Radiologist*	1.68	1.64	1.58	1.79	1.65	1.66	1.62	1.69	1.25
Model	1.34	1.41	1.38	1.37	1.47	1.46	1.44	1.51	1.45
P	0.08	0.19	0.23	0.01	0.34	0.21	0.24	0.29	0.16
C4/5									
Radiologist*	1.81	1.80	1.51	1.95	1.71	1.59	1.54	1.69	1.42
Model	1.71	1.68	1.51	1.64	1.77	1.60	1.67	1.89	1.65
P	0.58	0.54	0.99	0.13	0.77	0.96	0.43	0.32	0.19
C5/6									
Radiologist*	1.58	1.58	1.54	1.73	1.62	1.48	1.66	1.58	1.53
Model	1.74	1.80	1.44	1.75	1.72	1.54	1.69	1.70	1.57
P	0.41	0.25	0.60	0.87	0.56	0.75	0.86	0.51	0.80
C6/7									
Radiologist*	1.54	1.62	1.30	1.73	1.62	1.37	1.46	1.70	1.42
Model	1.42	1.27	1.41	1.40	1.30	1.35	1.40	1.34	1.28
P	0.51	0.02	0.45	0.04	0.03	0.88	0.72	0.02	0.33

*MAE* mean absolute error, *F/E* flexion–extension, *F/N* Flexion–neutral, *N/E* Neutral–extension

*P* < 0.05 indicates significant inter-observer difference

Radiologist* indicates the other radiologist

## Discussion

In the present study, we developed a deep learning-based model to detect landmarks necessary for measuring cervical SIRM. On the test set, we found that our landmark detection network achieved the PCKs ranging from 94 to 100% at the 2-mm distance threshold. Based on this network, the developed model for automatic SIRM measurements was comparable in performance to radiologists’ calculations.

Manual annotation is the main source of observer differences in spinal measurement. The reliability depends on the experience and judgment of radiologists. A landmark-to-landmark distance of 2.98 mm for inter-observer observations was reported to be acceptable for clinical analysis [26]. In the present study, the PCKs of our developed model at a 2-mm distance threshold ranged from 97 to 98% on the three views, which was similar to the percentage of inter-observer landmark distance from three radiologists. As a result, both the model and the radiologists were able to provide reliable landmark annotations. On all three views, the PCKs of C2 within a 2-mm distance threshold were relatively higher than other segments, which might be contributed to fewer degenerations occurring at C2 [27].

Measurement performance is mostly evaluated by calculating various measurement errors. In the applications of the spine, the MAEs could be less than 2° or close to 10° [19, 28]. This indicates the performance of models varying greatly in different spinal landmarks and parameters. For SIRM, the interobserver variability of manual measurement could be up to 5.2°, which might not provide accurate evaluations for instability, abnormality, and preoperative motion function [29]. A study conducted by Frobin et al. reported that the error (standard deviation, SD) of approximately 2° was satisfactory for clinical cervical motion analysis [21]. In our test set, the model demonstrated excellent measurement performance and reliable clinical application with MAEs and SDs ranging from 1.19° to 1.69° and 1.50° to 2.27°, respectively. In the comparison of the model and radiologists, the MAEs of our model were equal to or significantly lower than those of the radiologists, indicating that our model showed similar or smaller errors compared to the radiologists. The model also achieved satisfactory agreement from C3/4 to C6/7, but the ICCs and rs at C2/3 were not sufficient to guarantee reliable consistency, especially on N/E motion (ICC = 0.60, *r* = 0.61). This might be due to the extremely small range of motion at C2/3, resulting in even slight differences (MAE = 1.21–1.40) having significant impacts [20].

Flexion, neutral, and extension cervical lateral radiographs are essential tools in the assessment of cervical SIRM and there will be great translational potentials in future clinical practice. The model will automatically generate parameter measurement reports for doctors and patients to facilitate clinical diagnosis and treatment guidance. With the expansion of the database, the obtained measurement results will be used to build population-based models to provide personalized reference intervals for cervical SIRM of different genders and ages in asymptomatic and symptomatic individuals.

The present study does have several limitations. First, for the correctness and integrity of landmark annotation, we excluded a large number of patients based on postoperative status and obscuration of the C7 vertebral bodies. Second, because of inherent variations in manual annotation and the lack of a gold standard, some difficulties remain in accurately comparing performance between radiologists and the model. Third, the category and size of the training dataset are insufficient to represent the complex clinical environment. In future research, we will include more kinds of cases, with particular inclusion of patients with implanted surgical devices. For unsatisfactory landmark prediction due to anatomical variation or overlap, radiologists could slightly adjust the landmarks, and the feedback could be used to enhance model performance in efforts to further improve our model.

## Conclusions

A deep learning-based model was developed for automated SIRM measurement on flexion–neutral–extension cervical lateral radiographs and showed comparable performance with radiologists. It may provide rapid, accurate, and comprehensive information for cervical motion analysis.

## Supplementary Information

**Additional file 1.** Percentage of correct key points (PCK) for landmarks at the 1–5-mm thresholds on flexion, neutral, and extension views.

**Additional file 2.** Bland–Altman plots (left) and correlation scatter diagrams (right) showing the differences and correlations between the model and reference standards on F/N motion.

**Additional file 3.** Bland–Altman plots (left) and correlation scatter diagrams (right) showing the differences and correlations between the model and reference standards on N/E motion.

## Data Availability

The datasets generated and analyzed during the current study are not publicly available due to patient privacy concerns but are available from the corresponding author on reasonable request.

## Abbreviations

CNN: Convolutional neural network
CI: Confidence interval
F/E: Flexion–extension
F/N: Flexion–neutral
HRNet: High-Resolution Net
ICC: Intra-class correlation coefficient
r: Pearson correlation coefficient
RMSE: Root mean square error
SIRM: Sagittal intervertebral rotational motion
MAE: Mean absolute error
MPCK: Mean of the percentage of correct key points
N/E: Neutral–extension
PACS: Picture archiving and communication system
PCK: Percentage of correct key points
