# Supplementary material for: Sagittal intervertebral rotational motion: a deep learning-based measurement on flexion–neutral–extension cervical lateral radiographs
Source: BMC Musculoskelet Disord. 2022 Nov 8;23:967. doi: 10.1186/s12891-022-05927-0 (PMC9641900; doi:10.1186/s12891-022-05927-0)
Supplement: Supplementary file 1 — Additional file 1. Percentage of correct key points (PCK) for landmarks at the 1–5-mm thresholds on flexion, neutral, and extension views. [file 12891_2022_5927_MOESM1_ESM.docx]

**Additional file 1** Percentage of correct key points (PCK) for landmarks at the 1–5-mm thresholds on flexion, neutral, and extension views.

| Threshold |  | 1mm |  |  |  | 2mm |  |  |  | 3mm |  |  |  | 4mm |  |  |  | 5mm |  |
| --- | --- | --- | --- | --- | --- | --- | --- | --- | --- | --- | --- | --- | --- | --- | --- | --- | --- | --- | --- |
|  | F | N | E |  | F | N | E |  | F | N | E |  | F | N | E |  | F | N | E |
| B3 | 87 | 94 | 94 |  | 99 | 99 | 100 |  | 99 | 100 | 100 |  | 99 | 100 | 100 |  | 99 | 100 | 100 |
| B4 | 95 | 91 | 87 |  | 99 | 99 | 99 |  | 99 | 100 | 100 |  | 99 | 100 | 100 |  | 99 | 100 | 100 |
| **MC2** | **91** | **93** | **91** |  | **99** | **99** | **100** |  | **99** | **100** | **100** |  | **99** | **100** | **100** |  | **99** | **100** | **100** |
| C1 | 79 | 86 | 80 |  | 97 | 99 | 100 |  | 99 | 100 | 100 |  | 99 | 100 | 100 |  | 99 | 100 | 100 |
| C2 | 77 | 77 | 76 |  | 95 | 98 | 97 |  | 99 | 99 | 100 |  | 99 | 100 | 100 |  | 99 | 100 | 100 |
| C3 | 83 | 87 | 90 |  | 98 | 99 | 98 |  | 99 | 100 | 100 |  | 99 | 100 | 100 |  | 99 | 100 | 100 |
| C4 | 91 | 94 | 86 |  | 100 | 100 | 99 |  | 100 | 100 | 100 |  | 100 | 100 | 100 |  | 100 | 100 | 100 |
| **MC3** | **83** | **86** | **83** |  | **98** | **99** | **99** |  | **99** | **100** | **100** |  | **99** | **100** | **100** |  | **99** | **100** | **100** |
| D1 | 79 | 81 | 69 |  | 96 | 100 | 94 |  | 98 | 100 | 97 |  | 98 | 100 | 100 |  | 98 | 100 | 100 |
| D2 | 80 | 75 | 82 |  | 98 | 97 | 97 |  | 98 | 99 | 100 |  | 99 | 100 | 100 |  | 99 | 100 | 100 |
| D3 | 90 | 82 | 86 |  | 99 | 99 | 98 |  | 100 | 100 | 98 |  | 100 | 100 | 99 |  | 100 | 100 | 99 |
| D4 | 91 | 83 | 89 |  | 100 | 100 | 99 |  | 100 | 100 | 100 |  | 100 | 100 | 100 |  | 100 | 100 | 100 |
| **MC4** | **85** | **80** | **82** |  | **98** | **99** | **97** |  | **99** | **100** | **99** |  | **99** | **100** | **100** |  | **99** | **100** | **100** |
| E1 | 78 | 75 | 61 |  | 98 | 99 | 95 |  | 100 | 100 | 98 |  | 100 | 100 | 100 |  | 100 | 100 | 100 |
| E2 | 71 | 79 | 75 |  | 99 | 99 | 98 |  | 100 | 100 | 100 |  | 100 | 100 | 100 |  | 100 | 100 | 100 |
| E3 | 71 | 79 | 77 |  | 96 | 94 | 96 |  | 100 | 100 | 99 |  | 100 | 100 | 100 |  | 100 | 100 | 100 |
| E4 | 87 | 88 | 91 |  | 98 | 99 | 99 |  | 100 | 100 | 100 |  | 100 | 100 | 100 |  | 100 | 100 | 100 |
| **MC5** | **77** | **80** | **76** |  | **98** | **98** | **97** |  | **100** | **100** | **99** |  | **100** | **100** | **100** |  | **100** | **100** | **100** |
| F1 | 83 | 84 | 62 |  | 99 | 99 | 95 |  | 100 | 100 | 100 |  | 100 | 100 | 100 |  | 100 | 100 | 100 |
| F2 | 68 | 82 | 75 |  | 100 | 99 | 100 |  | 100 | 100 | 100 |  | 100 | 100 | 100 |  | 100 | 100 | 100 |
| F3 | 81 | 88 | 85 |  | 99 | 98 | 98 |  | 99 | 99 | 99 |  | 100 | 100 | 100 |  | 100 | 100 | 100 |
| F4 | 91 | 85 | 91 |  | 99 | 100 | 100 |  | 100 | 100 | 100 |  | 100 | 100 | 100 |  | 100 | 100 | 100 |
| **MC6** | **81** | **85** | **78** |  | **99** | **99** | **98** |  | **100** | **100** | **100** |  | **100** | **100** | **100** |  | **100** | **100** | **100** |
| G1 | 93 | 88 | 89 |  | 99 | 100 | 99 |  | 99 | 100 | 99 |  | 99 | 100 | 99 |  | 99 | 100 | 99 |
| G2 | 70 | 73 | 78 |  | 98 | 99 | 99 |  | 100 | 100 | 100 |  | 100 | 100 | 100 |  | 100 | 100 | 100 |
| G3 | 92 | 93 | 86 |  | 99 | 100 | 100 |  | 100 | 100 | 100 |  | 100 | 100 | 100 |  | 100 | 100 | 100 |
| G4 | 88 | 83 | 84 |  | 98 | 95 | 97 |  | 100 | 99 | 99 |  | 100 | 99 | 99 |  | 100 | 100 | 100 |
| **MC7** | **96** | **84** | **84** |  | **99** | **99** | **99** |  | **100** | **100** | **100** |  | **100** | **100** | **100** |  | **100** | **100** | **100** |
| **Total** | **81** | **83** | **80** |  | **97** | **98** | **97** |  | **99** | **100** | **99** |  | **99** | **100** | **100** |  | **99** | **100** | **100** |

F: flexion; N: neutral; E: extension.
