# Supplementary material for: Sagittal intervertebral rotational motion: a deep learning-based measurement on flexion–neutral–extension cervical lateral radiographs
Source: BMC Musculoskelet Disord. 2022 Nov 8;23:967. doi: 10.1186/s12891-022-05927-0 (PMC9641900; doi:10.1186/s12891-022-05927-0)
Supplement: Supplementary file 2 — Additional file 2. Bland–Altman plots (left) and correlation scatter diagrams (right) showing the differences and correlations between the model and reference standards on F/N motion. [file 12891_2022_5927_MOESM2_ESM.docx]

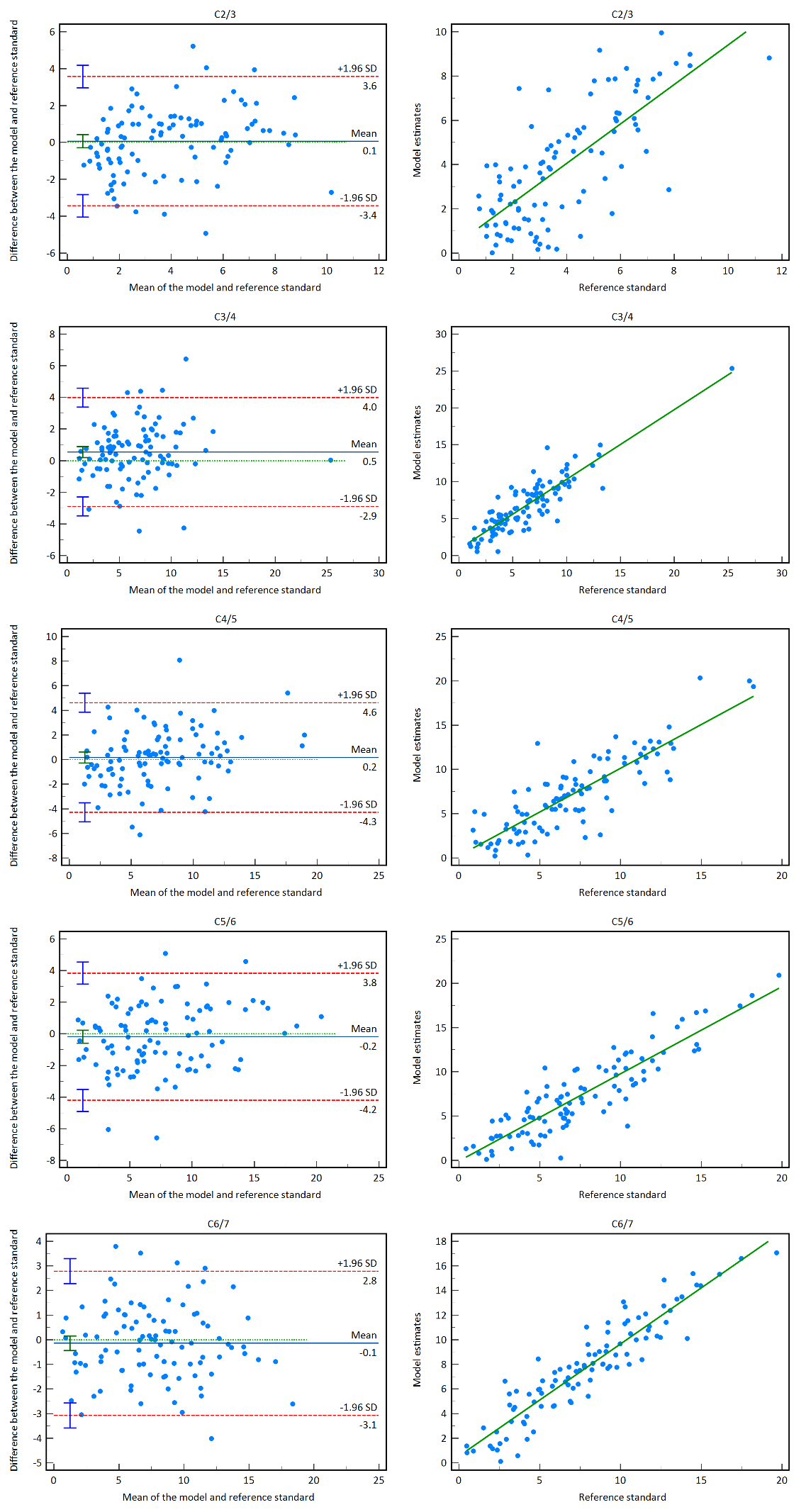


**Additional file 2** Bland–Altman plots (left) and correlation scatter diagrams (right) showing the differences and correlations between the model and reference standards on F/N motion.
